# Garlic Ideotypes for Future Korean Climates

**DOI:** 10.3390/plants15193002

**Published:** 2026-09-30

**Authors:** Nayoung Woo, Kyungdahm Yun

**Affiliations:** Department of Smart Farm, Jeonbuk National University, 567 Baekje-daero, Deokjin-gu, Jeonju 54896, Republic of Korea

**Keywords:** *Allium sativum*, crop modeling, virtual genotype, ideotype, climate change adaptation, Cropbox

## Abstract

Climate change may alter which garlic traits are most useful for crop improvement. We used Garlic.jl, a process-based garlic crop growth model, to evaluate combinations of 13 growth and developmental traits under parameter backgrounds for two cultivars, Namdo and Daeseo, across six sowing dates and 10 Korean sites. Simulations compared dry bulb yield rankings under current and future climates using a common harvest date. Many combinations ranked highly under the current climate lost position under the strongest future projection, although yield differences among leading candidates were often small. Under future climates, maritime simulations suggested slower leaf initiation and longer leaf expansion duration, southern mainland environments favored long leaves with slower leaf tip appearance, and northern environments favored faster leaf initiation with slower appearance. The two cultivar backgrounds produced similar overall rankings but differed in some leading trait combinations. These findings can guide regional garlic breeding by identifying trait combinations and planting strategies to test under future cultivation environments.

## 1. Introduction

Garlic growth is strongly controlled by the sequence of storage and field temperatures, photoperiod, and planting date [1,2,3]. Autumn-planted garlic establishes before winter, resumes rapid canopy growth in spring, forms bulbs as temperature and day length increase, and is harvested in early summer [2,4]. Exposure to low temperature promotes subsequent bulbing, whereas long days and warmer spring conditions accelerate bulb development and maturity [1,5,6]. The magnitude and timing of these responses vary among genotypes [7,8]. Planting date further changes the environmental sequence experienced during development [9]. Korean observations and warming experiments also link winter and spring conditions with garlic growth and production [10,11]. Climate change may therefore alter several connected developmental windows within one crop cycle.

This environmental sensitivity creates a breeding problem. Traits favored by the current production calendar may confer different benefits after warming, atmospheric drying, and rising carbon dioxide (CO_2_) concentrations [12,13]. Multi-environment garlic trials already show genotype-by-environment interaction for bulb yield and identify both broadly and specifically adapted materials [14,15,16]. Breeding new combinations is difficult because commercial garlic is propagated clonally and many cultivars are sterile [2,17]. Historical improvement has therefore relied heavily on introduced germplasm and clonal selection [17,18]. Restored fertility, genomic resources, and sexually derived families are expanding the available tools, but cultivar development and regional testing remain slow [19,20,21].

Korea provides a clear example of rapid change in the cultivar landscape. Korean garlic is commonly classified into warm-region and cold-region ecotypes, also called southern and northern types, according to winter adaptation and growth timing [22,23]. Namdo was selected from Chinese Gajungbaek material introduced in 1976 and released for warm southern regions in 1983 [24]. Daeseo originated from Spanish material grown in Changnyeong and was distributed as a very early cultivar in 1986 [24,25]. Both belong to the warm-region ecotype, but Daeseo generally emerges and matures earlier and produces larger bulbs and higher yield per unit area under comparable management [25,26]. Daeseo now accounts for more than half of the national garlic area, whereas the share planted to Namdo has declined substantially [27,28]. Official assessments associate this shift with yield, marketable bulb size, peeled garlic recovery, flavor, and mechanization [27]. Present commercial success, however, does not establish future climatic suitability.

Field experiments cannot reproduce late-century climates or evaluate hundreds of hypothetical cultivars before they exist. Crop models provide a complementary virtual experiment by connecting weather and management with the plant processes that determine growth and yield [29,30,31]. The first process-based Garlic.jl study focused on temperature-dependent development and the growth of individual leaves [4]. The model was later extended with canopy carbon assimilation and biomass allocation that depends on developmental stage to simulate bulb yield [23]. CropSyst has separately been applied to garlic dry matter, nitrogen uptake, and yield [32]. In onion, a closely related crop in the same genus, ALCEPAS represented potential crop growth and bulb formation, while subsequent models emphasized photothermal control of bulbing [33,34,35,36]. These models provide physiological tools for climate analysis, but virtual genotype selection has not, to our knowledge, been applied to *Allium* crops.

The ideotype concept frames breeding targets as coordinated plant characteristics suited to a defined environment [37]. Model-assisted ideotype studies have shown that productive trait combinations depend on the target environment. Virtual tomato canopies and numerical apple plants separated high productivity from biological attainability [38,39]. Future ideotypes in wheat, barley, rice, and sorghum varied among climate zones, model structures, and selection objectives [40,41,42,43]. A MAIZSIM study evaluated virtual maize trait and management combinations across the United States and found that both relative rankings and favorable traits changed between current and future climates [44]. Broader analyses likewise show that cultivar duration and sowing time can modify climate responses and that temperature, vapor pressure deficit, and CO_2_ should not be treated as interchangeable drivers [45,46,47]. Such predictions remain conditional on model structure and omitted stresses, so they are best used to define testable breeding hypotheses [48].

This study used a process-based garlic crop model with current and future climate data to address three questions. First, do virtual garlic genotypes favored under the current climate retain their relative rankings under future climates? Second, which traits and sowing dates become more favorable across contrasting production environments? Third, what do these changes imply for breeding and management when viewed alongside two widely cultivated Korean cultivars? The analysis compares relative performance and is not intended to predict absolute farm yields.

## 2. Materials and Methods

### 2.1. Study Design

**Figure 1:**
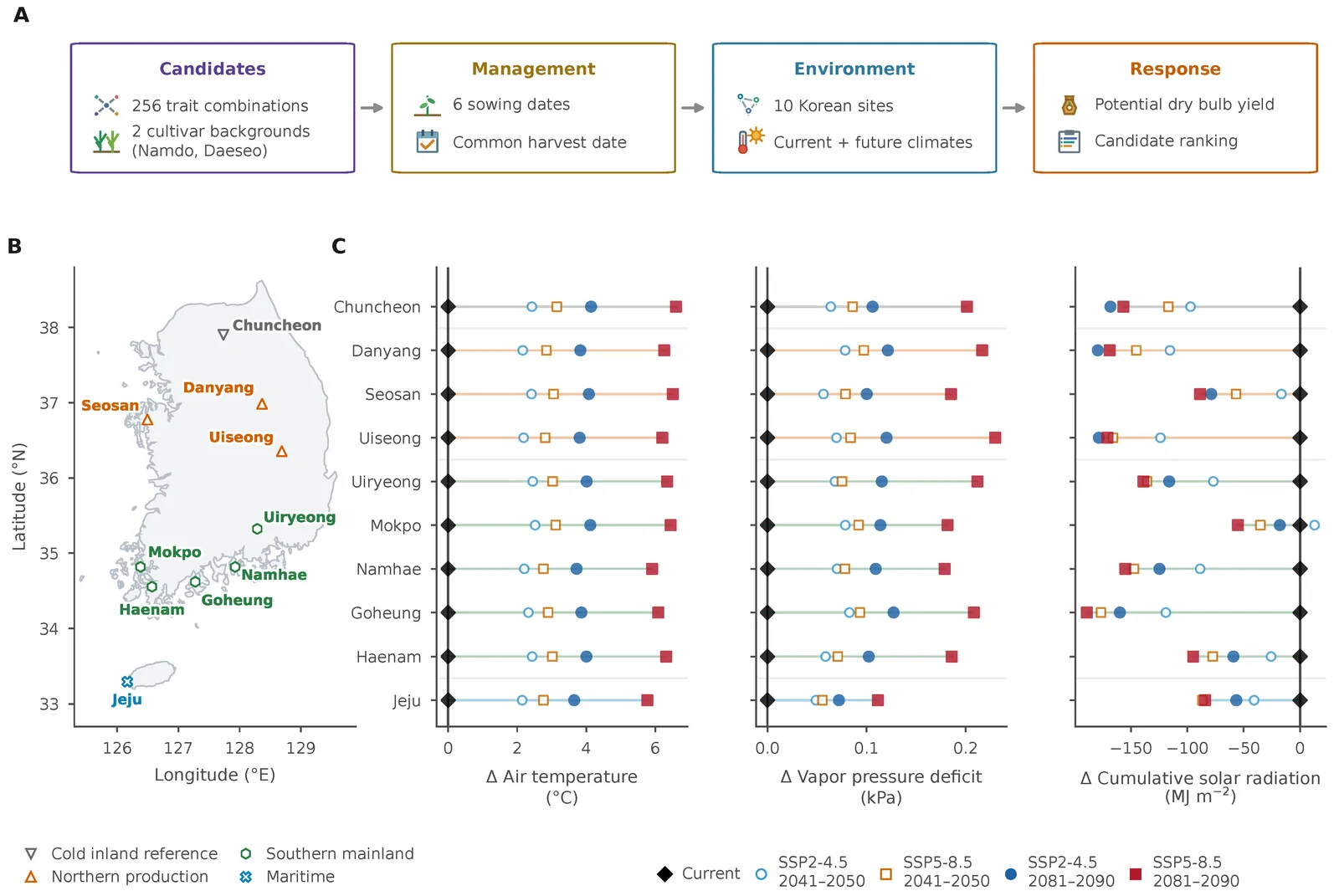
The study design and climate contrasts. Panel (**A**) summarizes the virtual trait combinations, cultivar backgrounds, sowing dates, climate inputs, and yield ranking. Panel (**B**) shows the 10 study sites. Jeju represents the maritime environment. Mokpo, Haenam, Goheung, Uiryeong, and Namhae form the southern mainland production target. Seosan, Uiseong, and Danyang form the northern production target, and Chuncheon is a cold inland reference outside the main production areas. Panel (**C**) gives crop season changes in air temperature, vapor pressure deficit, and solar radiation relative to the 1991–2020 climatological normal. All future site and climate combinations were warmer and had higher vapor pressure deficit. Solar radiation declined in 39 of 40 comparisons.

The design sampled 256 combinations of 13 traits. A current climate leaf number screen retained 164 combinations for evaluation at six sowing dates and 10 Korean sites under the 1991–2020 climatological normal and four future climate states (Figure 1A). Each retained combination was evaluated using parameter backgrounds for two cultivars, Namdo and Daeseo. The complete cultivar parameter sets were evaluated separately as cultivar references. Analyses focused on bulb dry yield rankings, traits of the top groups, regional responses, and changes in canopy development.

### 2.2. Model

The simulations used Garlic.jl 0.1.32, a process-based growth model for hardneck garlic developed with the Cropbox 0.3.52 framework [23,49]. Garlic.jl couples hourly development, canopy formation, and carbon balance. Temperature and photoperiod regulate leaf initiation, leaf tip appearance, and developmental transitions. Individual leaves follow rank-specific growth and senescence trajectories that determine the canopy available for radiation interception [4]. C_3_ gas exchange supplies assimilated carbon, which is reduced by respiration and allocated among vegetative and reproductive organs according to developmental stage. Bulb dry mass and surviving density determine potential dry bulb yield per unit ground area [23]. Soil water and nitrogen modules have not yet been integrated into this model configuration. The simulations therefore represent potential production without water or nutrient stress and do not predict farm yield or marketable bulb quality.

### 2.3. Virtual Genotypes

**Figure 2:**
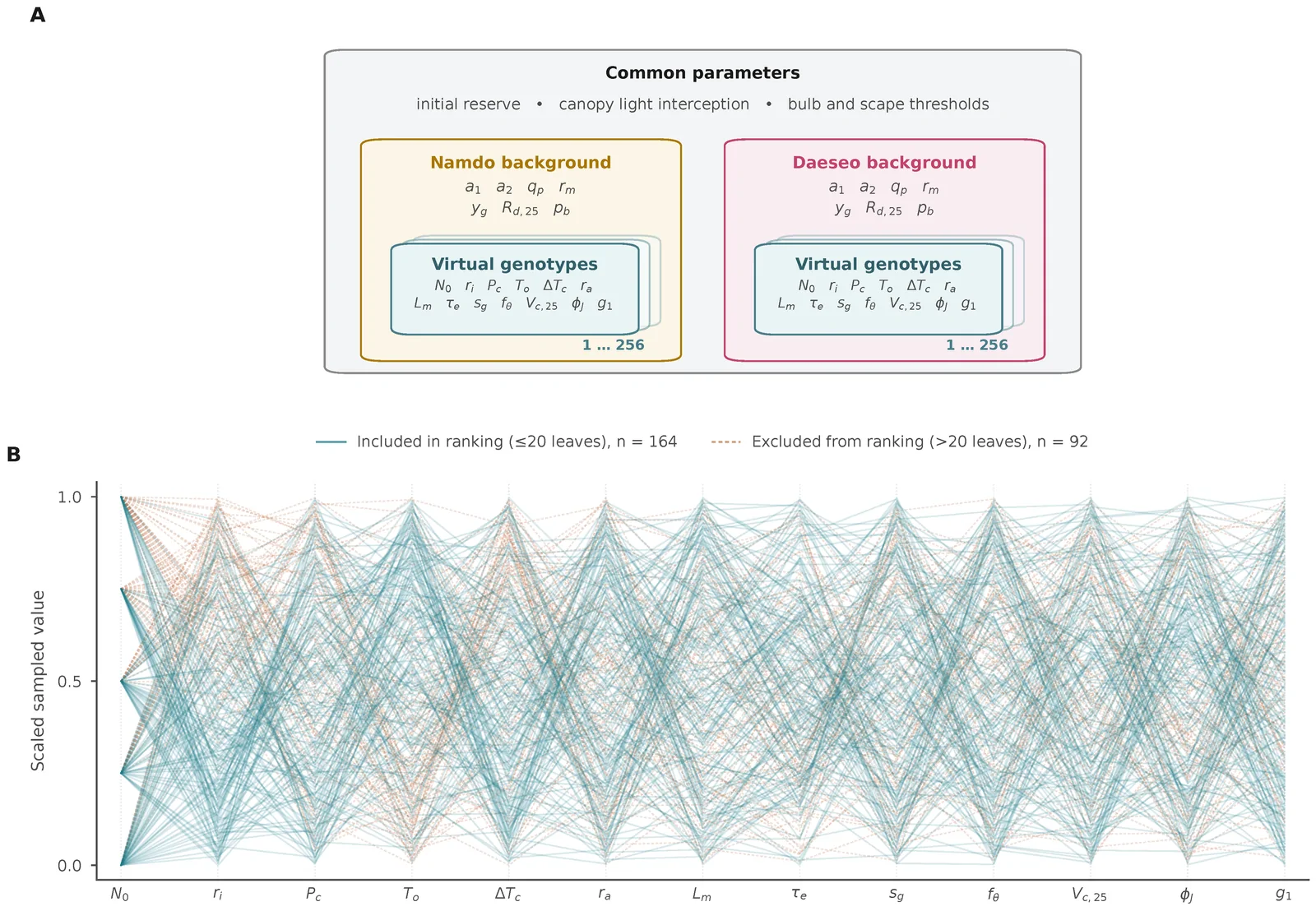
Construction and coverage of the virtual genotype population. Panel (**A**) shows how each of the same 256 sampled trait vectors was combined separately with the Namdo and Daeseo cultivar backgrounds. Panel (**B**) shows all vectors after scaling each trait to its sampled range. The primary ranking population contains the 164 vectors that produced no more than 20 appeared leaves at Jeju under the current climate. The other 92 were excluded from the primary ranking analysis. Trait symbols and ranges are defined in Table 1, and the principal background differences are listed in Table 2.

The virtual genotypes varied 13 traits describing development, canopy construction, gas exchange, and canopy persistence (Table 1). Each 13-trait combination was paired independently with both cultivar backgrounds. In each paired version, the sampled values replaced the same 13 entries while all other values remained those of Namdo or Daeseo, respectively. Here, background means the cultivar parameter set retained outside the 13 sampled traits. It does not denote pedigree, a simulated cross, or an estimated genetic background effect. These retained values included leaf allometry, leaf rank shape, respiration, carbon allocation, and other unsampled processes. The design therefore produced two paired versions of every virtual genotype (Figure 2).

A randomized Latin hypercube generated 256 combinations across the trait ranges without evaluating a complete grid [50]. We retained the space filling design with the greatest minimum distance among candidate designs. Feasible leaf growth constraints linked potential leaf length and expansion duration, while the other sampled traits retained broad coverage with little pairwise association. Initial leaf number was mapped to integers from four to eight. Maximum leaf elongation rate was derived from potential leaf length and expansion duration, electron transport capacity from carboxylation capacity and its sampled ratio, and ceiling temperature from the developmental optimum and the optimum to ceiling interval. The carboxylation range was centered on a published hardneck garlic estimate [51]. All parameters not represented in Table 1 retained their cultivar background or common default values. The sampled bounds defined an exploratory model space rather than an observed breeding population.

An initial screen retained 164 combinations that produced a cumulative appeared-leaf count of no more than 20 across the six sowing dates at Jeju under the current climate. Jeju was the warmest current site and produced the largest leaf counts. Both backgrounds gave identical screening results, and this population was held fixed across environments. The value of 20 was a permissive screening limit, not a biological ceiling. Alternative limits tested sensitivity to this choice (Section 2.12). Future profiles with more than 20 appeared leaves were retained to compare the same population across climates.

### 2.4. Cultivar Backgrounds

Namdo and Daeseo were selected as two widely cultivated warm-region ecotype cultivars in Korea. Representative parameter sets distributed with Garlic.jl were used as cultivar backgrounds. Within these parameter sets, Namdo had a longer potential leaf, lower maintenance respiration, higher synthesis efficiency, and distinct stage-specific carbon allocation. Daeseo had faster maximum rates of leaf initiation and leaf tip appearance, a shorter potential longest leaf, greater leaf area per unit length, and a higher senescence threshold (Table 2).

Each complete parameter set was also evaluated as a cultivar reference. Parameters not listed in Table 1 and Table 2 followed the assigned background or common model defaults. Neither background represents the cold-region ecotype. Northern sites instead tested possible future cultivation environments for warm-region garlic, following earlier projections of northward expansion under warming [23].

### 2.5. Management and Sites

Each parameter set was combined with sowing dates, meaning dates of clove planting, on days 250, 260, 270, 280, 290, and 300. These dates correspond approximately to 7 September through 27 October at 10-day intervals. This range spans current Korean planting recommendations for warm-region and cold-region garlic ecotypes [22,52]. The comparisons assumed sufficient chilling for subsequent bulbing. Preplant storage was represented at 8 °C from 30 June to planting, giving 69–119 days for the storage response in leaf development [4,23]. This setting was not a test of chilling fulfillment. Plant density was fixed at 50 plants m^−2^, scapes were removed when the simulated event occurred, and all crops were evaluated on 15 May of the following year, hereafter termed the common harvest date. An earlier garlic modeling study used 15 May to represent practical Namdo harvest timing in Jeju [23]. We retained this date as a common calendar reference across genotypes, sites, and climate states. It was not treated as physiological maturity or an optimal harvest date. Canopy peak and scape diagnostics were retained to identify mismatches between crop development and this common calendar.

The 10 locations represented maritime, southern mainland, northern production, and cold inland environments (Figure 1B, Table 3). The first three groups were regional selection targets. Chuncheon was retained as a cold inland reference. Production locations were chosen from designated garlic regions and to span contrasting ecotypes and planting calendars [22,53].

### 2.6. Climate Inputs

Site-specific climate values were extracted from approximately 30 m Agricultural Digital Climate Maps (AgDCMs) supplied directly by the National Center for Agro-Meteorology (NCAM) [54]. The 1991–2020 climatological standard normal, hereafter the current climate, was based on Korea Meteorological Administration (KMA) observations. Future inputs represented Shared Socioeconomic Pathway (SSP) scenarios SSP2-4.5 and SSP5-8.5 in 2041–2050 and 2081–2090. They were based on the ensemble mean of five regional climate models (CCLM, GRIMs, HadGEM3-RA, RegCM, and WRF) driven by the UK Earth System Model, version 1 (UKESM1) [54]. Future anomalies relative to 1981–2010 were applied to observation-based maps for that period to adjust model bias [55]. Topoclimatic methods provided spatial detail [56,57]. Each supplied climate state contained 365 daily climatological values of minimum, mean, and maximum air temperature and solar radiation. These represented the seasonal cycle averaged over the respective 30-year or 10-year period, not a sequence of individual years [54]. A valid solar radiation pixel 0.28 km from the Jeju site coordinate was used because that coordinate fell outside the coastal raster.

Daily values were converted to the hourly inputs required by Garlic.jl. Hourly temperature followed a smooth daytime rise and nighttime decline while retaining the daily minimum, mean, and maximum [58]. Solar radiation was distributed over daylight hours while preserving its daily total. Actual vapor pressure was approximated from minimum temperature, and hourly relative humidity was calculated following Food and Agriculture Organization (FAO) guidance for missing humidity data [59]. Wind speed was fixed at 2 m s^−1^ for leaf boundary-layer conductance in the gas-exchange module [60].

Atmospheric CO_2_ was supplied separately from weather. Current simulations used 390 μmol mol^−1^ [23], and future concentrations came from the harmonized SSP dataset [61]. Because a garlic season spans two calendar years, the model received the sowing-to-harvest mean of the annual concentrations, rounded to the nearest μmol mol^−1^. Primary simulations paired each weather state with its corresponding CO_2_.

### 2.7. Ranking Analysis

Ranking analysis compared bulb dry yield by the common harvest date across representative climate states, rather than final yield at matched physiological maturity. Each virtual genotype combined with one sowing date was treated as a candidate profile, giving 984 profiles per environment and cultivar background. Profiles were ranked by bulb dry yield within each site and climate state. Regional profile scores were the arithmetic mean of those within-site ranks. Averaging ranks prevented sites with larger yields from dominating regional selection. Genotype scores were the mean profile rank across sites and the six sowing dates. Lower scores indicated better performance. All rankings were calculated independently within each background.

Spearman rank correlation measured agreement between current and future rankings. A coefficient of 1 indicates identical ordering, a value near 0 indicates little consistent association, and a value of −1 indicates reversal. Absolute rank movement and retention of current top 10 members described changes near the selection boundary. Tied yields received average ranks. The primary top 10 used candidate identifiers to resolve tied regional scores, and a sensitivity comparison also included candidates tied at the cutoff.

### 2.8. Trait Analysis

Trait directions were summarized in two ways. Spearman rank correlations related each trait to yield after averaging across sowing dates. Medians, interquartile ranges, and full ranges described the 10 genotypes with the highest ranks. Profile summaries described sowing date, canopy development, and phenology. These correlations were associations within the sampled design rather than independent genetic effects or a global sensitivity analysis. Canopy persistence refers to the senescence threshold multiplier $s_{g}$. Model diagnostics included peak leaf area index, appeared leaf number, stand survival, scape occurrence, and timing of maximum green leaf area. Normalized green leaf area duration was the seasonal integral of green leaf area divided by its maximum. Future extrapolation was assessed without changing the fixed population. Selected profiles above 20 appeared leaves were counted, and trait summaries were repeated using only genotypes that remained at or below that threshold throughout each regional comparison.

### 2.9. Background Comparison

The background comparison tested whether unsampled cultivar parameters changed the selected combinations. The paired candidates differed only in the parameters retained from Namdo or Daeseo. Agreement was summarized by rank correlation, top 10 overlap, winner identity, and changes in top group trait medians. These comparisons tested sensitivity within Garlic.jl and did not represent pedigree or crosses.

### 2.10. Cultivar References

The complete cultivar parameter sets were also evaluated in the southern target. Their best sowing dates and potential yields were followed across climates. Each cultivar was compared with virtual profiles evaluated on its corresponding background. Fixed comparison groups were selected separately within each background and contained the 10 profiles ranked highest under the current climate or late-century SSP5-8.5. Rankings were never mixed across the two backgrounds. Cultivar yield was also expressed relative to the mean of the state-specific top 10 virtual profiles in the corresponding background.

### 2.11. Climate Drivers

Crossing current and future weather with reference and future CO_2_ across the complete experiment separated their effects on potential yield and rankings. Weather combined temperature, vapor pressure deficit, and solar radiation. Regional percentage responses used mean yields across genotypes and sites relative to the corresponding current baseline, both within each sowing date and after averaging across dates. The interaction was the joint response minus the separate weather and CO_2_ effects. Rank correlation and top 10 overlap tested CO_2_ effects under identical weather, using regional mean yield within each sowing date and the primary mean rank objective across all profiles.

To examine the large yield responses, hourly assimilation and maintenance respiration were integrated for four trait combinations representing the 10th, 50th, and 90th percentiles of current southern yield pooled across backgrounds and the leading southern profile. These diagnostics used both backgrounds, all sites, sowing day 270, and current and late-century SSP5-8.5 climates through the common harvest date.

### 2.12. Sensitivity Analysis

Sensitivity analysis tested the influence of harvest timing, leaf number screening, and selection rules. Simulations continued through 3 July and were evaluated weekly from 24 April, comparing current and future rankings on matching dates. Harvest tests used the primary population, both backgrounds, and all sites. The main comparison used 8, 15, and 22 May, while later dates assessed continued growth rather than marketable maturity. To relate yield performance to canopy size and persistence, we used cumulative green leaf area duration, which is the integral of green leaf area from planting to harvest. Spearman correlations assessed whether greater green leaf area duration was associated with better yield rankings and whether future increases accompanied rank improvement.

Leaf number screening limits of 18, 20, 22, and 24 were applied under current Jeju conditions, and each population was evaluated across all sites, backgrounds, and climate states. These screens differed from the future leaf number restrictions in Section 2.8.

Selection groups of 5, 10, 20, and 50 candidates and approximately 5% and 10% of each population were compared. Direct regional mean yield provided an alternative to mean rank. Yield margins between leading profiles and gains from selecting a new top group described the magnitude of differences behind rank changes. These deterministic contrasts were not statistical confidence intervals or validated breeding gains.

## 3. Results

### 3.1. Climate

Every projected crop season became warmer and had greater atmospheric drying, while nearly every projection also received less solar radiation (Figure 1C). Across sites and future states, mean temperature increased by 2.14–6.60 °C and mean vapor pressure deficit increased by 0.049–0.230 kPa. Cumulative solar radiation declined in 39 of 40 comparisons. The only increase was 13 MJ m^−2^ at Mokpo under mid-century SSP2-4.5. The future forcing therefore combined faster accumulation of thermal time and greater evaporative demand with slightly less incident energy.

The regional climate gradient remained recognizable. Jeju was the warmest and least atmospherically dry environment, the southern mainland was warm with the greatest crop season radiation, and the northern production sites and Chuncheon were cooler. Under late-century SSP5-8.5, regional mean warming ranged from 5.77 °C in Jeju to 6.60 °C in Chuncheon. The cooler environments therefore warmed slightly more, but they did not become equivalent to the southern or maritime environments. These contrasts supported separate regional summaries. The projections describe deterministic climate trajectories rather than the frequency of extreme years.

### 3.2. Ranking Changes

**Figure 3:**
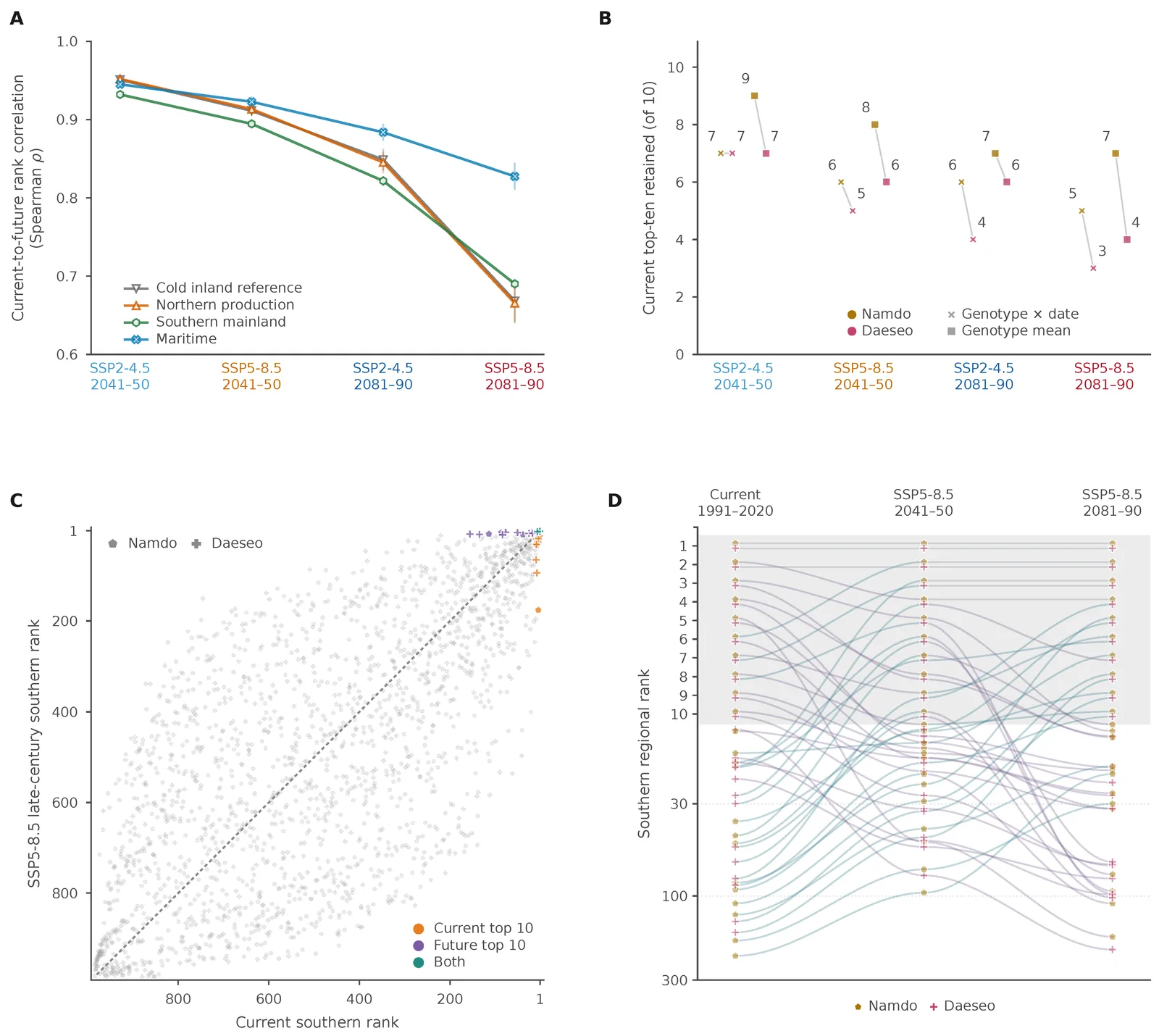
Changes in candidate rankings from the current climate to four future states after independent evaluation in the Namdo and Daeseo backgrounds. Panel (**A**) gives Spearman rank correlation between the current and future candidate profiles in the four regional environments. Each marker is the mean of the two background coefficients, and the thin vertical segment spans their values. Panel (**B**) gives the number of current top 10 members retained in the corresponding future top 10. A profile is one genotype and sowing date combination among 984 candidates. A genotype is one of 164 trait vectors scored by averaging its profile ranks across the six sowing dates and constituent sites. Panel (**C**) overlays the southern ranks for each background under the current climate and late-century SSP5-8.5. Panel (**D**) follows every profile that entered a southern top 10 under the current, mid-century SSP5-8.5, or late-century SSP5-8.5 climate. Ranks 1–10 have equal spacing, and lower positions are logarithmically compressed.

Figure 3 summarizes changes in ranking agreement, top group retention, and individual rank paths.

Agreement between current and future rankings weakened with the severity and time horizon of the climate state in both cultivar backgrounds (Figure 3A). For the 984 southern profiles defined by genotype and sowing date, the Namdo and Daeseo correlations were 0.937 and 0.927 under mid-century SSP2-4.5, 0.900 and 0.889 under mid-century SSP5-8.5, 0.828 and 0.815 under late-century SSP2-4.5, and 0.691 and 0.690 under late-century SSP5-8.5. The corresponding numbers of current top 10 profiles retained were 7 and 7, 6 and 5, 6 and 4, and 5 and 3 (Figure 3B). Under late-century SSP5-8.5, the median absolute movement was 148 ranks with Namdo and 142 with Daeseo. Their 90th percentiles were 391 and 390 positions (Figure 3C). The broad loss of ranking persistence was therefore reproducible across backgrounds, while retention at the selection boundary remained background dependent.

**Figure 4:**
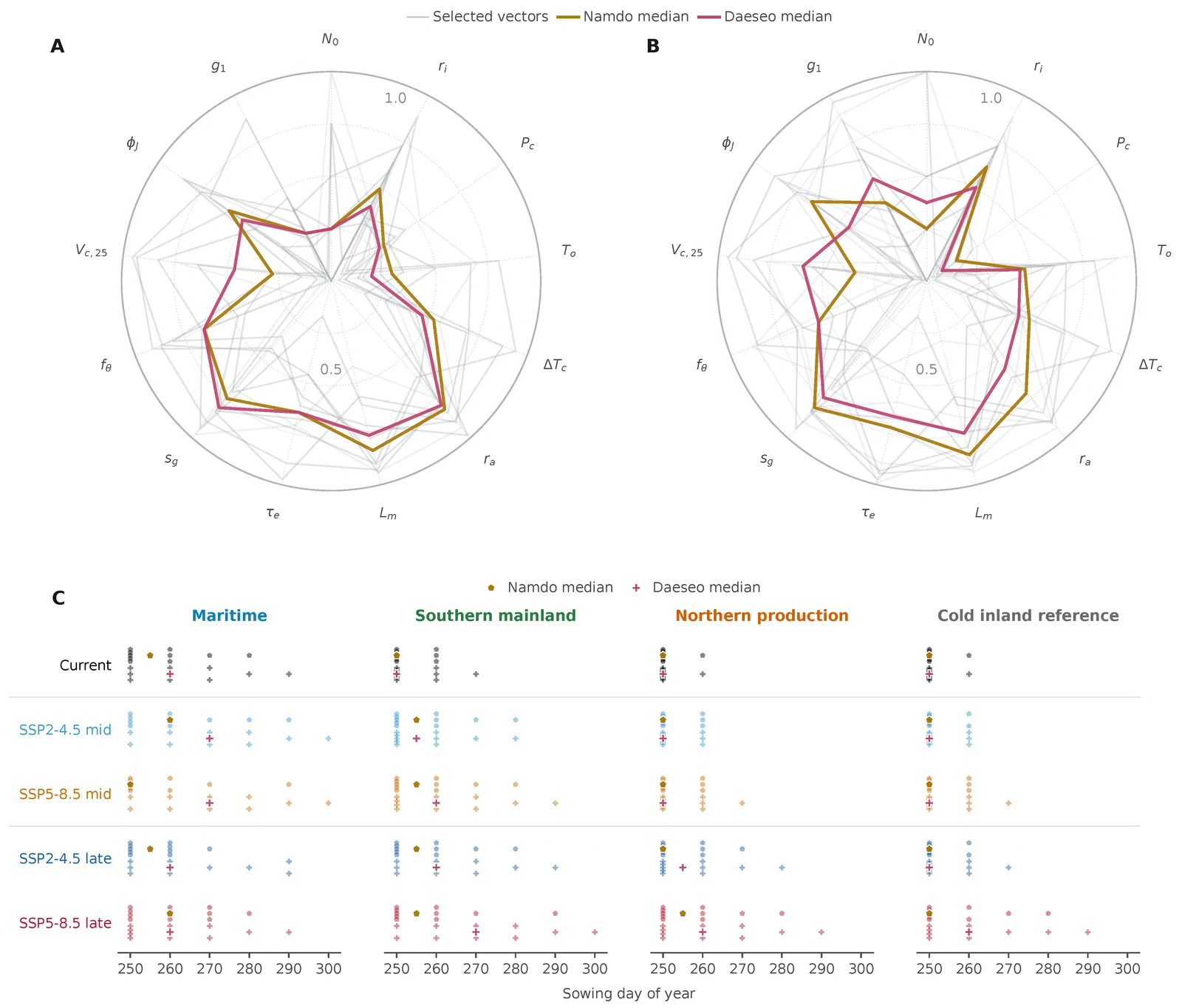
Trait composition and sowing dates of the virtual candidates with the highest ranks in both cultivar backgrounds. Panels (**A**,**B**) show the southern top 10 genotypes under the current climate and late-century SSP5-8.5, respectively. The light lines show all selected vectors, and the heavier lines show the median for each background. Values are scaled from zero to one using the sampled bounds in Table 1. Panel (**C**) shows the sowing dates of all 10 profiles with the highest ranks in each background and climate state for the maritime, southern mainland, northern production, and cold inland reference environments, from left to right. Current, mid, and late denote 1991–2020, 2041–2050, and 2081–2090, respectively. $N_{0}$, $r_{i}$, $P_{c}$, $T_{o}$, and $\Delta T_{c}$ denote initial leaf number, maximum leaf initiation rate, critical photoperiod, developmental optimum temperature, and the interval from optimum to ceiling temperature. $r_{a}$, $L_{m}$, $\tau_{e}$, $s_{g}$, and $f_{\theta }$ denote maximum leaf tip appearance rate, potential longest leaf length, leaf expansion duration, senescence threshold multiplier, and leaf angle factor. $V_{c,25}$, $\phi_{J}$, and $g_{1}$ denote maximum carboxylation capacity at 25 °C, the maximum electron transport to carboxylation ratio, and stomatal slope.

Genotype scores averaged profile ranks across sowing dates and showed a similar pattern (Figure 3B). The paired Namdo and Daeseo correlations were 0.940 and 0.930, 0.903 and 0.896, 0.833 and 0.824, and 0.704 and 0.708 across the four future states. The corresponding top 10 retention counts were 9 and 7, 8 and 6, 7 and 6, and 7 and 4. These counts used a less stringent selection fraction than the profile top 10 and do not by themselves establish greater genotype stability. Sowing date changed the position of individual genotypes (Figure 3D). Current top profiles used days 250–270, whereas late-century SSP5-8.5 top profiles spanned days 250–300 (Figure 4C). The wider future range indicates a flatter timing response for some genotypes rather than a universal shift to one later optimum. In the southern target, one virtual genotype sown on day 250 ranked first under both backgrounds in every climate state (Figure 3D and Table 4).

The maritime environment retained more similar rankings across the full candidate set but showed sharper turnover within the top 10 (Figure 3A,B). At Jeju, late-century SSP5-8.5 profile correlation was 0.810 with Namdo and 0.844 with Daeseo, yet only one and zero current top 10 profiles remained. The northern production correlations were 0.690 and 0.641, with five and two retained. Chuncheon gave 0.697 and 0.640, also with five and two retained. In the northern production target, the current winner differed between backgrounds, but the same day-250 profile ranked first in both under late-century SSP5-8.5. Agreement across all candidate ranks and retention within the top 10 therefore captured different aspects of ranking change.

In every southern comparison, the candidates ranked tenth and eleventh had different mean rank scores, so the primary groups and the groups retaining all cutoff ties were identical. Exact cutoff ties occurred in a northern profile comparison under mid-century SSP2-4.5 and a Chuncheon genotype comparison under late-century SSP2-4.5. Including both tied candidates expanded those two sensitivity groups to 11 members, without changing the regional interpretation.

### 3.3. Trait Profiles

**Figure 5:**
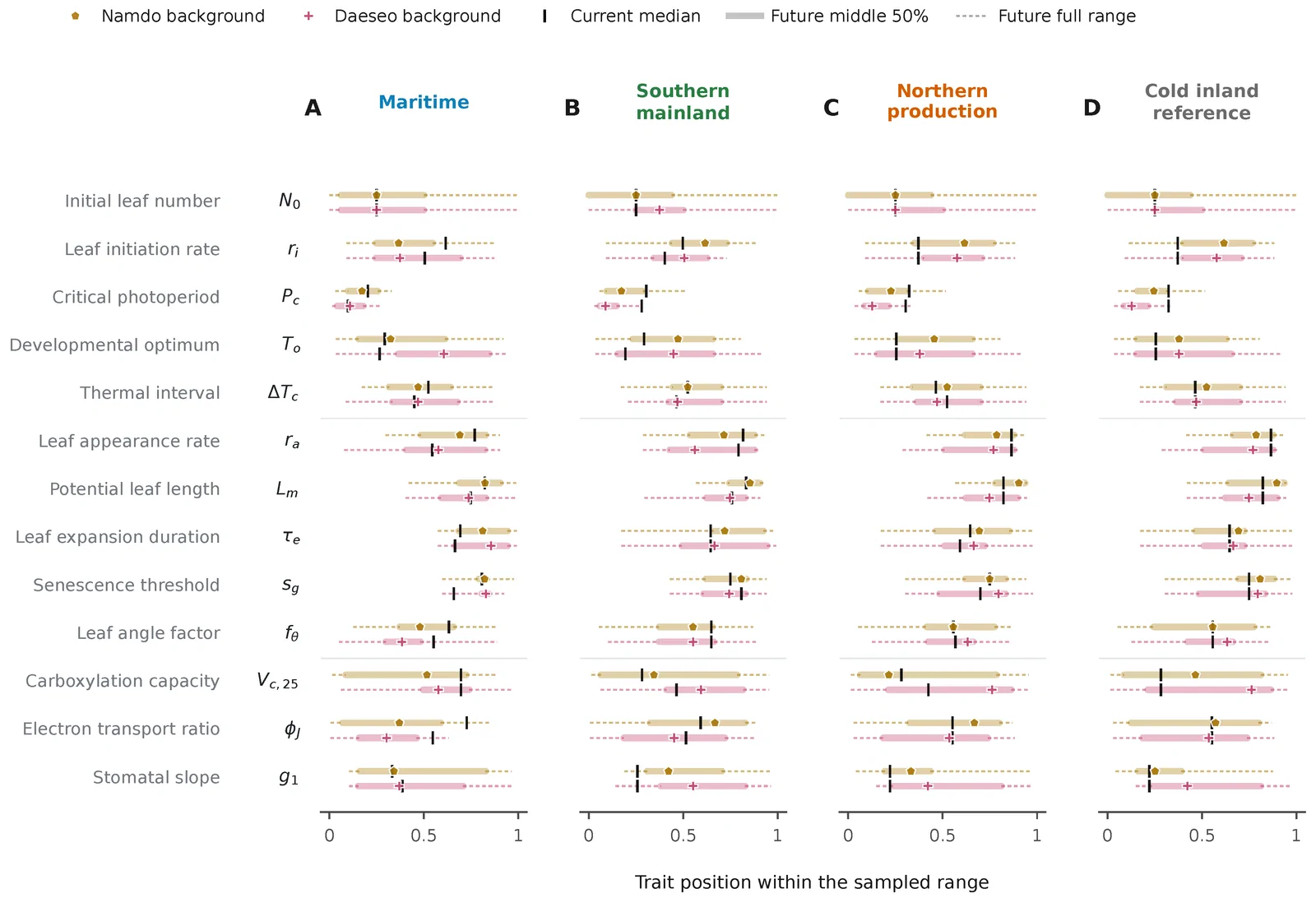
Regional trait composition of the top 10 virtual genotypes under the current climate (1991–2020) and late-century SSP5-8.5 (2081–2090). Panels (**A**–**D**) show the maritime, southern mainland, northern production, and cold inland reference environments. For each cultivar background, symbols show future medians and vertical ticks show current medians. Thick segments enclose the middle 50% of future top 10 trait values, and dashed extensions show their full range. These are distributions within the selected groups, not confidence intervals. Values are normalized to the sampled bounds in Table 1, with zero and one representing the lower and upper bounds. Symbols summarize selected virtual genotypes, not the complete cultivar parameter sets. The comparison shows regional differences in preferred trait levels and their changes from the current climate.

The genotypes ranked highest were defined by coordinated trait combinations rather than one extreme (Figure 4A,B). Trait balances differed among regions (Figure 5A–D). In the maritime environment, leaf initiation slowed by 0.017 d^−1^ with Namdo and 0.009 d^−1^ with Daeseo, while leaf expansion lengthened by 1.66 and 2.67 d. Leaf tip appearance slowed slightly with Namdo but accelerated slightly with Daeseo.

In the southern mainland, both backgrounds selected slower leaf tip appearance, a lower critical photoperiod, a warmer developmental optimum, and longer leaf expansion under late-century SSP5-8.5. The changes with Namdo and Daeseo were respectively −0.056 and −0.127 d^−1^ for leaf tip appearance, −0.33 and −0.48 h for critical photoperiod, +0.81 and +1.15 °C for developmental optimum, and +1.03 and +0.27 d for expansion duration. Potential longest leaf length remained in the upper part of the sampled range but changed by +1.26 cm with Namdo and −0.62 cm with Daeseo. Canopy persistence also changed in opposite directions, increasing by 0.18 with Namdo and decreasing by 0.19 with Daeseo. The central developmental directions were therefore shared, whereas the exact leaf size and persistence combination depended on the retained cultivar parameters (Figure 5B).

In the northern production target, both backgrounds instead selected faster leaf initiation, slower leaf tip appearance, and longer expansion. In this northern target, median sowing date moved from day 250 under the current climate to day 255 with Namdo and day 260 with Daeseo under late-century SSP5-8.5, although the selected ranges were broad (Figure 4C). At the cold inland reference, the corresponding late-century medians were day 250 with Namdo and day 260 with Daeseo.

Spearman rank correlations across all 164 genotypes supported several top group shifts. In the southern mainland, the correlations for potential leaf length increased from 0.498 to 0.582 with Namdo and from 0.507 to 0.527 with Daeseo. Leaf expansion duration increased from 0.216 to 0.391 and from 0.228 to 0.380, while canopy persistence increased from 0.337 to 0.613 and from 0.348 to 0.587. The critical photoperiod correlations became more negative in both backgrounds. By contrast, the developmental optimum correlations weakened from approximately −0.26 to −0.03. The warmer optimum of the future top group was therefore a property of selected multivariate combinations rather than a strong marginal association. This contrast is visible among the selected genotypes with high ranks. The current southern leader after averaging across sowing dates had $T_{o}=20.6$ °C, whereas the future leader had $T_{o}=23.6$ °C (Table 4). Carboxylation capacity also showed only a weak association with simulated yield and did not dominate rankings within the sampled range.

The future leaf number diagnostic qualified these selected groups. Under late-century SSP5-8.5, three of the ten southern profiles with the Namdo background and two with the Daeseo background exceeded 20 appeared leaves. At Jeju, the corresponding counts were eight and five. These profiles extended beyond the initial screening range. None of the northern production or Chuncheon top 10 profiles exceeded 20 leaves, and none of the selected profiles exceeded 24. Requiring all six sowing dates to remain within the 20-leaf threshold at every constituent site retained 124 of 164 southern genotypes and 80 of 164 Jeju genotypes. Within this restricted set, slower leaf tip appearance, lower critical photoperiod, and a warmer optimum remained in both southern backgrounds. Expansion duration increased by 0.79 d with Namdo and decreased by 0.30 d with Daeseo. The main southern directions were therefore not wholly dependent on extrapolated profiles, whereas expansion duration and the strongest maritime combination were less robust.

The leading day-250 profile had a mean peak leaf area index of 3.50 across the five southern sites under the current climate and 5.14 under late-century SSP5-8.5. The future increase extends beyond the conditions used to develop the cultivar parameter sets, so the selected profiles remain breeding hypotheses that require canopy measurements.

### 3.4. Background Dependence

Applying the same trait vectors to the two cultivar backgrounds produced similar broad rankings but did not preserve every candidate at the selection boundary. Under the current southern mainland climate, between-background rank correlation was 0.977 for the 984 profiles and 0.976 for the 164 genotypes after averaging across sowing dates. Seven profile members and nine genotype members were shared between the two current top tens. Under late-century SSP5-8.5, the corresponding correlations were 0.973 and 0.974, while six profiles and seven genotypes were shared. One day-250 profile ranked highest in both backgrounds under the current climate and all four future states. After averaging across sowing dates, a different genotype led under the current climate, while the genotype underlying that persistent profile led under every future state in both backgrounds.

The two backgrounds also produced very similar rankings in the northern comparison, but agreement was weaker among candidates near the top of the ranking. The profile correlation between backgrounds was 0.990 under the current climate and 0.968 under late-century SSP5-8.5. All 10 current top profiles were shared, although the winner differed by background. Seven future top profiles were shared, and the same day-250 profile ranked first with both. At Jeju, only seven current and six late-century profiles were shared despite correlations of 0.966 and 0.985. Similar rankings across the full candidate set therefore did not guarantee the same leading local combinations.

Climate-driven rank shifts were strongly aligned between backgrounds, with ρ=0.981 for southern profiles under late-century SSP5-8.5. This agreement did not remove the background-dependent differences in profile and genotype retention at the top 10 boundary reported in Section 3.2.

Several regional trait directions were shared, but the combinations differed (Figure 5). Both maritime backgrounds selected slower initiation and markedly longer expansion. Both southern backgrounds selected a lower critical photoperiod, a warmer developmental optimum, slower leaf tip appearance, and longer leaf expansion. Canopy persistence increased from 3.25 to 3.42 with Namdo but decreased from 3.42 to 3.23 with Daeseo. Both northern backgrounds selected faster leaf initiation with slower leaf tip appearance. At Chuncheon, potential longest leaf length changed in opposite directions between backgrounds. This cold inland response was less robust than the results for production targets.

### 3.5. Cultivar References

**Figure 6:**
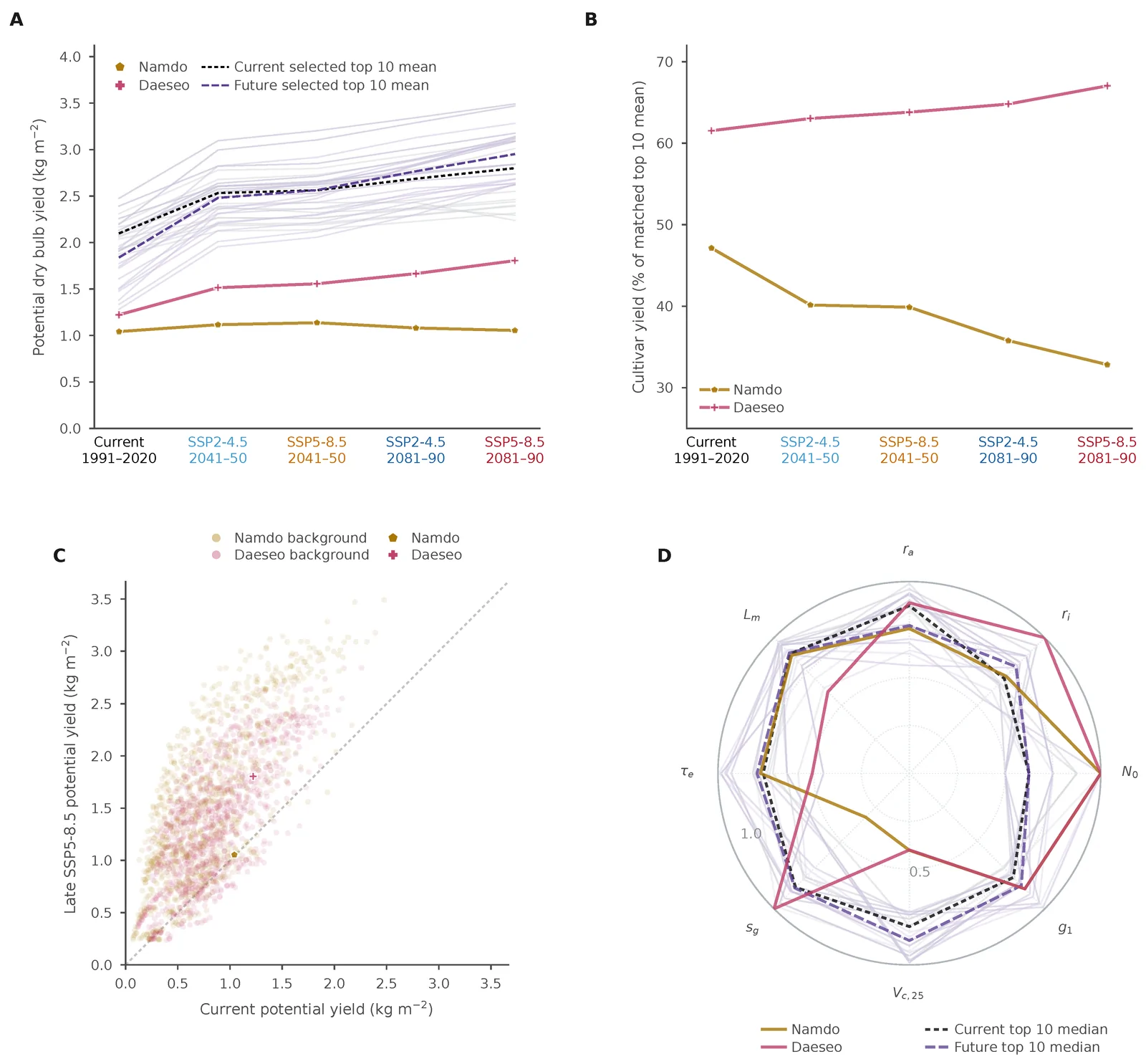
Representative Namdo and Daeseo parameter sets evaluated with the virtual genotypes. Panel (**A**) gives mean potential dry bulb yield across the five southern sites at the best cultivar sowing date in each climate state. The light trajectories and their means follow fixed top 10 cohorts selected separately within each matching background under the current climate or late-century SSP5-8.5. Panel (**B**) expresses each cultivar yield as a percentage of the mean of the state specific top 10 virtual profiles in its matching background. Panel (**C**) compares current and late-century SSP5-8.5 potential yield for all 984 virtual profiles in each background. Points above the diagonal increased under the future climate. Panel (**D**) compares eight traits chosen to summarize leaf development, canopy persistence, and gas exchange. It shows each complete cultivar parameter set together with individual vectors and pooled medians of the current and late-century top 10 genotypes for each background. Cultivar $\tau_{e}$ was derived as $L_{m}/r_{e}$. Each axis begins at zero, and its outer ring is the larger of the sampled upper bound and the two cultivar values. $N_{0}$, $r_{i}$, $r_{a}$, $L_{m}$, $\tau_{e}$, $s_{g}$, $V_{c,25}$, and $g_{1}$ denote initial leaf number, maximum leaf initiation rate, maximum leaf tip appearance rate, potential longest leaf length, leaf expansion duration, senescence threshold multiplier, maximum carboxylation capacity at 25 °C, and stomatal slope.

The complete cultivar parameter sets were evaluated separately from the paired background experiment (Figure 6). Namdo had a current southern mainland mean potential dry yield of 1.042 kg m^−2^ at sowing day 250. Day 250 remained its best sowing date except under late-century SSP2-4.5, when day 280 was favored. Under late-century SSP5-8.5, potential yield reached 1.054 kg m^−2^, an increase of 1.1%.

Daeseo remained best at day 250 in every climate state. Its modeled potential yield increased from 1.223 to 1.806 kg m^−2^ between the current climate and late-century SSP5-8.5, a 47.7% increase. Under the current common model conditions, the ratio of Daeseo to Namdo dry yield was 1.173. The direction agreed with a published field comparison, but differences in harvest, moisture basis, and management prevent interpreting this agreement as validation of the simulated magnitude [26].

The canopy and allocation diagnostics explained the contrasting absolute responses within the model. These diagnostics were averaged across the five southern sites at sowing day 250, which was the best date for both cultivars in the current and late-century SSP5-8.5 climates. Daeseo peak green leaf area increased by 41.6% and normalized green leaf area duration increased from 126.8 to 136.0 d. Its final bulb-to-total dry mass ratio changed from 48.7% to 44.4%. Namdo peak green leaf area increased by 18.7% and normalized duration increased from 104.4 to 108.3 d. Its total dry mass increased by 47.8%, but the bulb-to-total ratio decreased from 57.0% to 38.7%, leaving bulb dry yield nearly unchanged at the common harvest date. Modeled bulb appearance remained at 196 d after sowing in both climates, while Namdo’s mean appeared leaf number increased from 17.2 to 20.8 and scape appearance advanced by only 6 d. Because Namdo allocated smaller fractions to the bulb before and after scape appearance than Daeseo (Table 2), much of the additional dry matter remained in non-bulb organs by the common harvest. This simulated allocation reversal is therefore conditional on the developmental stage rules and common harvest date.

The background matched virtual cohorts provided context for the cultivar responses. In both backgrounds, the current top 10 gained absolute potential yield under late-century SSP5-8.5 but fell from a mean rank of 5.5 to approximately 27. Relative to the state-specific top 10 mean, Namdo decreased from 47.1% under the current climate to 32.8% under late-century SSP5-8.5. Daeseo increased from 61.5% to 67.0%. The virtual genotypes were assigned higher carboxylation capacity than either complete cultivar reference, representing hypothetical improvement in potential leaf photosynthetic capacity (Table 1 and Table 2). Their yield differences therefore reflect the sampled parameter ranges as well as canopy and allocation responses, rather than an established breeding gap.

### 3.6. Climate Drivers

**Figure 7:**
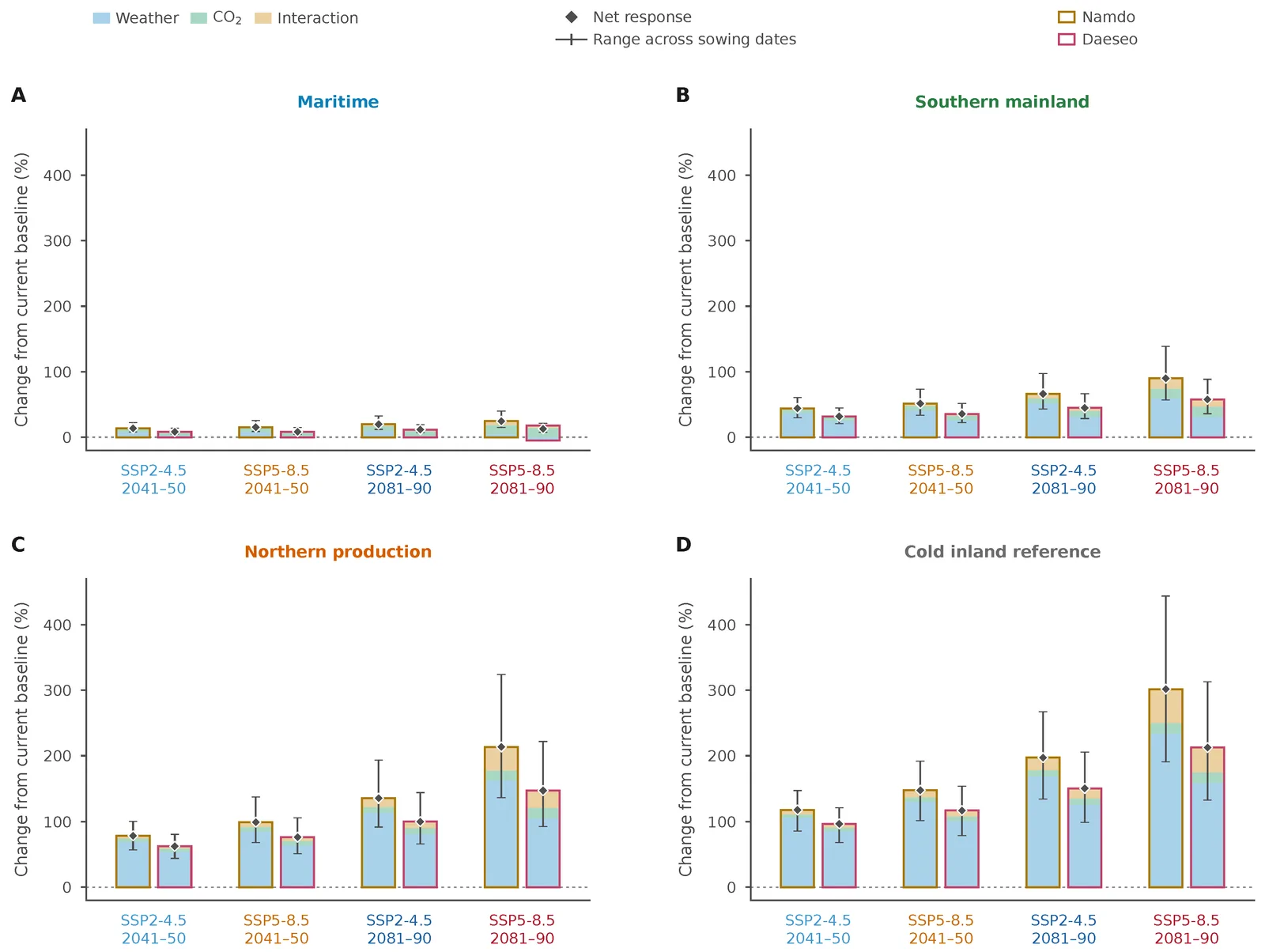
Effects of weather change, rising CO_2_, and their interaction across all six sowing dates in the Namdo and Daeseo backgrounds. Panels (**A**–**D**) show the maritime environment, southern mainland mean, northern production mean, and cold inland reference. Paired bars summarize potential yield on 15 May averaged across 164 trait combinations, regional sites, and sowing days 250–300 at 10-day intervals. Components are percentage changes relative to the corresponding mean yield under current weather and reference CO_2_. Their sum is the net response. Zero denotes no change, and a 100% increase doubles the current baseline yield. Vertical ranges show minimum and maximum net percentage responses among sowing dates, each relative to its own current baseline. They are not confidence intervals. Weather combines temperature, vapor pressure deficit, and solar radiation. Responses depend on sowing date, while maritime yields depend more on CO_2_ relative to weather.

Future weather and CO_2_ increased mean potential yield in every regional comparison after averaging across sowing dates (Figure 7). Under late-century SSP5-8.5, yield increased by 13–25% in the maritime environment and 58–90% in the southern mainland, while reaching 3.1–4.0 times the low current baseline at Chuncheon. Rising CO_2_ offset a negative average maritime weather response with Daeseo, whereas the combined weather response contributed more than CO_2_ in the south (Figure 7A,B). Sowing date changed response magnitude and even the sign of maritime weather effects. Larger percentage increases at later sowing partly reflected lower current yields, not higher future yields.

At sowing day 270, northern yield increased from 0.417 to 1.305 kg m^−2^ with Namdo and from 0.506 to 1.227 kg m^−2^ with Daeseo under late-century SSP5-8.5. Peak leaf area index increased from 1.15 to 2.16 and from 1.73 to 3.16, respectively. In the four-combination subset, northern gross assimilation increased by factors of 3.7 and 3.2, while maintenance respiration increased by approximately six and five, respectively. Survival changes contributed much smaller multipliers of approximately 1.03 in northern production environments and 1.06 at the cold inland reference. The proportion of Namdo profiles reaching peak green leaf area on the harvest date fell from 15–65% to 2–14% across sites. These responses reflected different developmental states on the same date, not validated changes in final marketable yield.

CO_2_ changed yield magnitude much more than relative rankings. Under identical future weather, genotype rank correlations were 0.9985–0.9999, with 9 or 10 top members retained across all 192 comparisons. Ranking genotype and sowing date profiles together gave correlations above 0.9992 and 8 to 10 retained profiles. Thus, CO_2_ occasionally changed membership near the selection boundary, while the broader ranking changes arose mainly from the combined weather response.

### 3.7. Sensitivity Analysis

**Figure 8:**
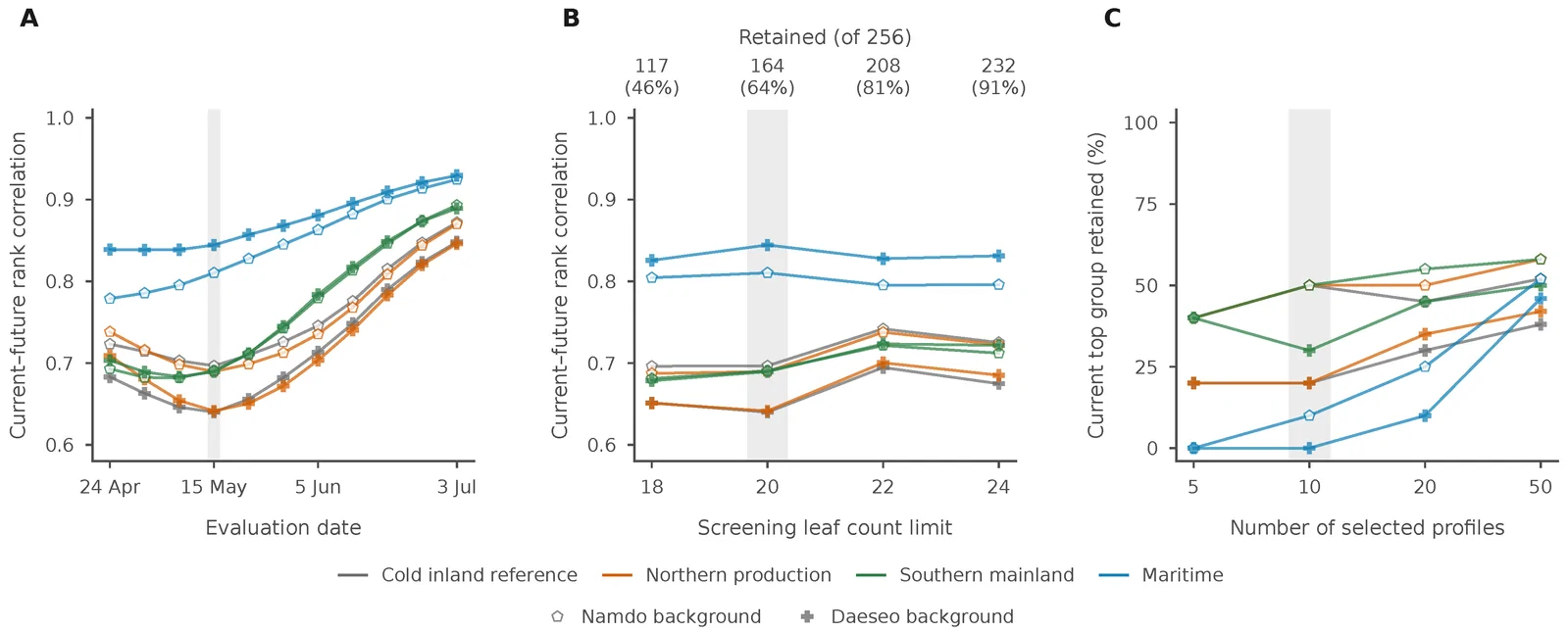
Sensitivity of candidate rankings to evaluation date and selection rules under current and late-century SSP5-8.5 climates. Panel (**A**) compares profile rankings on matching evaluation dates, with Spearman correlations nearer one indicating greater similarity. Panel (**B**) varies the leaf count limit for the current Jeju screen and evaluates the retained population at all 10 sites. Upper labels give the retained count and percentage of the 256 sampled vectors. Panel (**C**) gives the percentage of current selected profiles retained under future climate as the selection group is enlarged, using the primary 164-vector population. Shading marks the primary choices of 15 May in A, 20 leaves in B, and 10 profiles in C. Panels (**B**,**C**) use the 15 May evaluation. Broad ranking changes persist across these alternative choices.

Ranking changes and slower southern leaf tip appearance persisted across 8, 15, and 22 May in both backgrounds (Figure 8A). Other directions were conditional. Namdo’s developmental optimum shift and Daeseo’s expansion duration shift reversed between 15 and 22 May. Namdo’s southern yield response at its best sowing date changed from a 9.7% increase on 8 May to a 3.1% decrease on 22 May, while Daeseo increased by 44.1–51.7%. Later evaluation produced more similar rankings without establishing harvest suitability.

At 15 May under late-century SSP5-8.5, cumulative green leaf area duration was positively associated with yield performance across all four regions and both backgrounds (Spearman ρ=0.74–0.91). Its proportional increase from the current climate was also associated with rank improvement (ρ=0.68–0.85).

Broad ranking changes and slower southern and northern leaf tip appearance persisted across initial limits of 18–24 leaves, although some photoperiod and expansion shifts changed direction (Figure 8B). The limit of 18 leaves excluded the persistent winner. Enlarging the selected group did not eliminate replacement (Figure 8C). Mean yield and mean rank objectives agreed closely, with profile correlations above 0.9999, genotype correlations of at least 0.997, and 9 or 10 shared top members.

**Figure 9:**
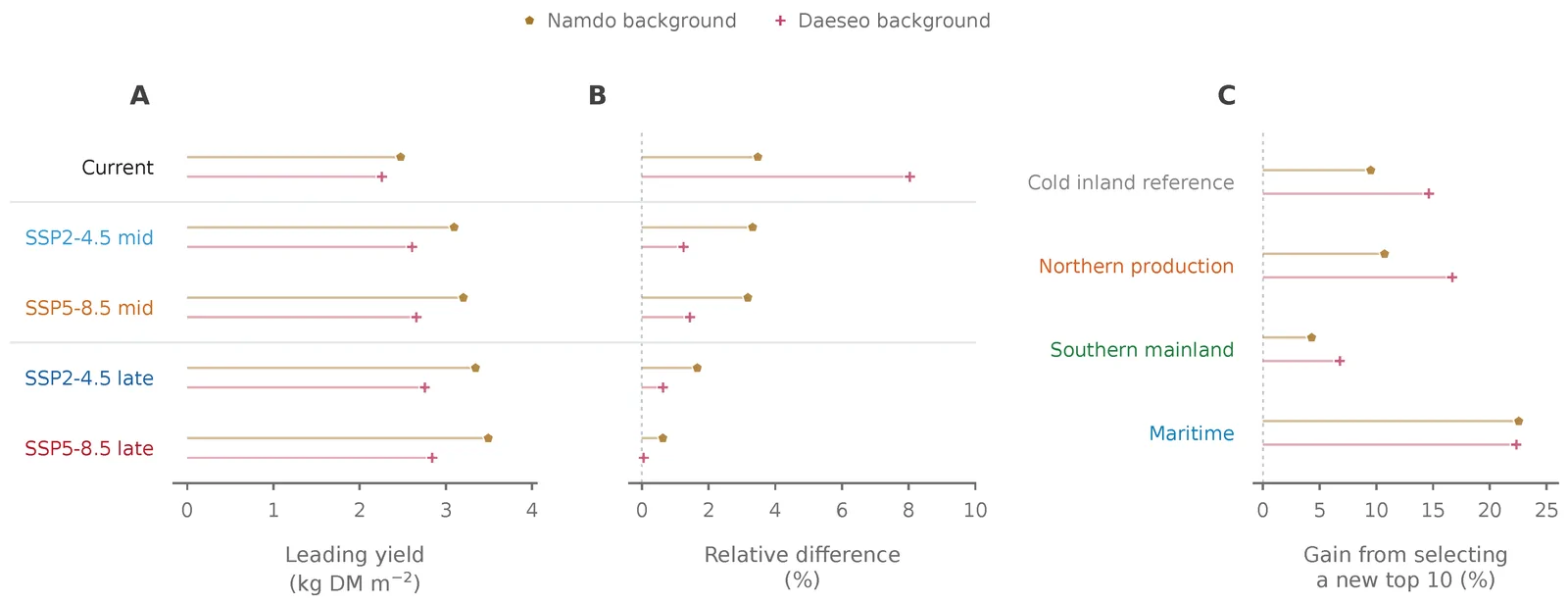
Yield differences among leading candidates. Panels (**A**,**B**) describe the leading southern mainland profile, which combined the same trait vector with sowing day 250 in every climate state and both backgrounds. Current, mid, and late denote 1991–2020, 2041–2050, and 2081–2090, respectively. Panel (**A**) shows mean bulb dry matter (DM) yield across the five southern sites. Panel (**B**) expresses its yield difference from the profile ranked second relative to that profile’s yield. Panel (**C**) compares mean yield of a newly selected future top 10 with that of the current top 10, both grown under late-century SSP5-8.5 in each region. Its percentage gain is relative to the latter group. All panels use the common 15 May harvest. Rank changes and persistence can coexist with small yield advantages.

Selecting a new southern top 10 increased late-century SSP5-8.5 yield by 4.3% with Namdo and 6.8% with Daeseo relative to retaining the current top 10 under the same future climate (Figure 9C). These gains were smaller than elsewhere, despite substantial rank movement. The persistent winner’s future margin over second place was only 0.63% with Namdo and 0.05% with Daeseo (Figure 9B). Rank changes and persistence therefore coexisted with small yield differences. The larger maritime selection gain requires caution because many selected profiles exceeded the initial leaf limit.

## 4. Discussion

### 4.1. Changing Rankings

Current suitability was informative but not durable enough to serve as the sole breeding target. Rankings across the full candidate set remained correlated under mid-century climates, yet turnover within the top 10 was substantial and increased under late-century SSP5-8.5. This distinction matters because breeding programs select from the upper tail. Studies of crop design likewise show that preferred combinations change with environment and selection objective [40,43,44,62]. Garlic trials have identified both broadly and specifically adapted clones through genotype-by-environment interaction [14,15]. Although those trials do not validate the simulated rankings, they support environment-dependent relative performance as biologically plausible.

The leading day-250 profile illustrates both the possibility and limits of ranking persistence. It ranked first for the southern mainland mean in every climate state with both backgrounds. Leaf length, leaf initiation rate, thermal breadth, leaf angle, and carboxylation capacity were near the upper ends of their sampled ranges, while expansion duration and canopy persistence were near their medians (Table 4). Yet its late-century margins of 0.63% with Namdo and 0.05% with Daeseo are too small to support practical superiority without uncertainty estimates (Figure 9B). The profile remained the southern winner on 8–22 May, but another profile led under the future climate by 5 June. It was also excluded by the tighter initial limit of 18 leaves. Its persistence is consequently conditional on the population and production calendar. Under the Namdo background at Jeju, it fell from rank 1 to 102. These results support examining a balanced group of combinations, not promoting a single universal optimum.

Day 250 is approximately 7 September. This date lies at the early edge of the usual September to early October window for garlic grown in warm southern regions and Jeju and precedes the October window recommended for cold-region garlic [22]. Within a cold-region garlic field trial limited to 20 October through 20 November, earlier sowing increased yield and marketable yield [52]. These observations do not mean that continued warming will shift every region toward earlier sowing. Experiments at elevated temperature suggested delayed sowing or harvest to reduce secondary clove formation, and an earlier Garlic.jl application projected later optimal planting at Jeju as the climate warmed while early September remained favorable at Chuncheon [11,23]. The present experiment likewise identified day 250 as a robust option for one balanced profile but found future top groups spanning through day 300. The practical hypothesis is therefore a planting window tailored to cultivar and region rather than a universal recommendation for earlier sowing. Joint adjustment of traits affecting the crop cycle and planting time has similarly changed projected climate adaptation in maize [63].

The paired backgrounds strengthened this interpretation while exposing its boundary. Rank shifts across the full candidate set were nearly identical, but the exact top 10 differed. Seven genotypes with the Namdo background and four with the Daeseo background remained in the top group between the current and strongest future climate. In the Namdo background, 8 of the 10 late-century top profiles belonged to only three genotypes. Several selected profiles can therefore represent alternative sowing dates for the same genotype. Selection fractions and yield margins provide essential context for top group turnover (Figure 8C and Figure 9C). Neither agreement between backgrounds nor persistence at nearby harvest dates establishes robustness to untested climate models, years, or genetic variation.

Absolute production and relative suitability must also be separated. The virtual population gained potential yield under every future climate, yet many current top combinations lost their position because others gained more (Figure 6C). The cultivar references showed the same distinction. A positive modeled production response can therefore coexist with a change in the preferred genotype.

### 4.2. Climate-Specific Traits

In the model, leaf appearance, potential leaf length, elongation, and senescence jointly determine the canopy trajectory [4,23]. The associations of cumulative green leaf area duration with yield performance and rank improvement are consistent with a role for canopy growth and persistence within the common production calendar (Section 3.7). Field defoliation studies also show that loss of green leaf area can reduce bulb yield, with strong effects around bulb initiation or several weeks before maturity [64,65].

The maritime target showed a distinct trait balance. Its unrestricted top group favored slower leaf initiation, substantially longer expansion, and greater canopy persistence, while critical photoperiod changed little. However, eight members with the Namdo background and five with the Daeseo background in the late-century top 10 exceeded the current 20-leaf threshold. This result therefore requires stronger qualification than the southern or northern results, although its sharp turnover supports treating Jeju separately. Temperature and photoperiod jointly regulate garlic bulbing, and experimental warming alters emergence and bulb development [5,7,11].

Long leaves with slower leaf tip appearance were the most consistent southern combination across the tested backgrounds, initial screens, and nearby harvest dates. This does not mean that warming slowed development. Selection favored a lower intrinsic maximum appearance rate within the warmer environmental sequence. The result suggests testing the timing of canopy construction, rather than maximizing development rate alone.

Other directions were more conditional. In the primary experiment, a lower critical photoperiod allowed floral initiation, the transition preceding bulb allocation, at shorter spring day lengths. However, the Namdo shift disappeared when the initial leaf limit was relaxed. This photoperiod parameter is not a measure of chilling requirement. A warmer developmental optimum occurred among some future top groups but showed weak marginal association and changed direction at a later harvest. These associations neither identify an independent genetic effect nor demonstrate a beneficial interaction. Expansion duration also depended on harvest date and on excluding future profiles above 20 leaves. The joint trait patterns are hypotheses for targeted measurements, not a formal global sensitivity analysis.

The northern environments selected faster leaf initiation with slower leaf tip appearance in both backgrounds across all four initial screening limits. Initiation of primordia and appearance of their tips are separate processes, so this combination can maintain leaf supply while spreading visible canopy construction over time. Longer expansion was less consistent when the screen was relaxed. These regions currently favor cold-region garlic, but warming may expand cultivation opportunities for warm-region cultivars [23]. Our northern comparisons address that possible expansion rather than simulate the cold-region ecotype itself. The results suggest that improvement of the same cultivar background may require different trait targets in established southern areas and prospective northern environments. Field tests would need to confirm winter survival and bulb development in those environments. Chuncheon’s opposite shifts in longest leaf length between backgrounds reinforce the importance of local evaluation.

Across regions, the modeled combinations are coordinated hypotheses rather than prescriptions to maximize one trait, consistent with environment-dependent trait value in other crops [45,66].

Canopy persistence illustrates the importance of trait combinations and retained background parameters. Its positive yield association strengthened under late-century SSP5-8.5 in both backgrounds, but the top 10 median increased with Namdo and decreased with Daeseo while remaining high in both. Similar canopy duration can arise from different combinations of leaf appearance, size, expansion, and senescence. Functional stay-green must also be distinguished from cosmetic chlorophyll retention [67]. Stay-green alleles in sorghum, for example, alter canopy development and the timing of water use rather than simply delaying leaf death [68]. Since the Garlic.jl multiplier only delays modeled green area loss, it does not independently represent chlorophyll retention or sustained photosynthetic capacity and must be interpreted with the other canopy and carbon balance traits.

Temperature, vapor pressure deficit, solar radiation, and CO_2_ act through different processes. Across the complete sowing window and four future states, rising CO_2_ increased potential yield but barely changed relative rankings when weather was held constant. Most ranking change therefore reflected the combined weather response and its interaction with traits. The magnitude of the yield response was less consistent across sowing dates. Daeseo showed smaller combined responses than Namdo, and its late-century Jeju weather effect was negative on average but became positive at the latest sowing date. A favorable net response can therefore mask an unfavorable weather component and cannot identify a universally favorable planting date. Vapor pressure deficit can affect yield independently of temperature, and the benefit of elevated CO_2_ depends on crop and water regime [46,47]. This study separated the complete weather bundle from CO_2_ but did not isolate individual weather variables.

### 4.3. Breeding Translation

Published field observations identify other Korean cultivars worth phenotyping against the modeled traits. Hongsan, a distinct cultivar, had longer leaves and later bolting than Daeseo. The cold-region cultivars Uiseong and Dansan had still longer leaves and later bolting with fewer visible leaves [26]. Hongsan also retained green leaves longer than Namdo and continued accumulating bulb yield with delayed harvest [69]. Its broad adaptation and late maturity make it a useful comparator for leaf size, canopy duration, and phenology [70]. These comparisons come from published field observations, not simulations of these cultivars, and do not by themselves establish slow maximum leaf appearance or functional stay-green.

Cold-region cultivars offer complementary traits rather than direct replacements for warm-region garlic. Their later development and stronger chilling requirement must be considered alongside useful canopy characteristics [22,70]. Uiseong and Dansan are candidates for studying long leaves and later development. Breeding for earlier maturity within this ecotype, as reported for Dansan and Gichan, also provides material for testing escape from adverse conditions late in the season [71,72]. Extending cultivar measurements and the relevant developmental processes would allow these materials to enter future model comparisons, broadening the choice between canopy persistence and earlier completion of growth.

Neither complete cultivar reference matched the overall trait profile of the future virtual top group (Figure 6D). Namdo retained approximately the same absolute potential yield and fell farther behind its matching frontier under the strongest future state. Daeseo gained potential yield and narrowed its relative gap, but it still remained below the future top group. These results do not identify a superior named cultivar. Such inference requires common measurements of leaf development, functional canopy persistence, gas exchange, allocation, and bulb yield across years and locations.

Translation also depends on whether the selected trait combinations can coexist in one plant. The virtual design sampled model parameters across bounded ranges and did not impose genetic correlations. Ideotype studies that constrained searches with observed phenotypic covariance or germplasm-specific parameter distributions produced more biologically achievable and sometimes different optima [39,73]. Garlic germplasm shows both extensive variation and repeated associations among leaf, plant, bulb, and reproductive traits [21]. The late-century top group should therefore be treated as a multivariate phenotyping target. Its individual parameter values should not be assembled independently as if every combination were genetically attainable.

The sampled model parameters are not direct field measurements, but they can be estimated from repeated observations. Maximum leaf tip appearance rate, $r_{a}$, can be fitted from tagged plant records of the dates when successive leaf tips become visible across planting dates and temperatures. The senescence multiplier, $s_{g}$, can be fitted from senescence dates for each leaf rank and repeated green leaf area or leaf area index trajectories. Weekly records of garlic leaf emergence, maturation, and senescence combined with destructive leaf measurements have already supported this type of parameterization [4]. Seasonal canopy light transmittance provides a scalable complement for estimating leaf area index [74].

Garlic breeding is constrained by vegetative propagation, frequent sterility, and variable compatibility [18]. Families produced from true seed nevertheless vary in bulb, leaf, stature, and maturity traits [19,20,75]. Crop models can support adaptation and be combined with genomic prediction in major crops [31,76], but garlic lacks validated genetic mappings for the physiological parameters used here despite growing genomic resources [21]. Phenotyping under a common protocol and fertility screening must therefore precede model-to-marker translation.

Fertile accessions can be considered separately from cultivars that carry desired canopy traits. Three garlic accessions in the USDA germplasm collection have useful reproductive characteristics. Their Plant Introduction accession numbers are PI 540316, PI 540319, and PI 540335. PI 540316 produced seed, while PI 540319 and PI 540335 showed high pollen viability or pollen tube development [75]. The male fertile line G398 also carries a characterized fertility associated marker [77]. None has yet been shown to combine fertility with the target canopy and phenology traits. A practical sequence is therefore to phenotype locally adapted cultivars and fertile accessions, create compatible segregating families, and select for the target canopy trajectory while retaining regional adaptation and bulb quality.

### 4.4. Cultivar Concentration

Daeseo first exceeded half of Korea’s garlic area in 2023, although this cultivar group statistic does not imply genetically identical planting stock [28]. Production and market evidence explains the shift more directly than the climate experiment. Official assessments cite yield, marketable bulb size, peeled garlic recovery, flavor, and mechanization [27], and a trial in a common environment reported more large bulbs and modestly higher cured yield [26]. Field statistics, cured yield, and modeled dry biomass differ in moisture basis, density, harvest, stand loss, and marketability [26,78]. The modeled dry yield contrast is therefore not independent cultivar validation.

Nevertheless, the cultivar references were not represented by a single yield offset. Their complete parameter sets retained differences in leaf development, leaf allometry, respiration, synthesis efficiency, and developmental carbon allocation that were specific to each cultivar (Table 2). These mechanisms produced the same current yield ordering as the available field comparison in a common environment and provided a limited physiological consistency check.

The model cannot by itself explain Daeseo expansion, and its future response does not establish climate superiority. Commercial dominance and future suitability are distinct objectives. Genotype-by-environment interaction in garlic supports testing broader diversity rather than assuming that present dominance implies future adaptation [14,15,16]. More broadly, declining diversity in cultivar responses to weather has raised concern about climate resilience in European wheat [79]. For garlic, maintaining and evaluating cultivars with contrasting developmental responses could preserve adaptation options alongside present market advantages.

### 4.5. Input Constraints

Garlic harvest timing reflects crop maturity as well as regional harvest windows extending from May into June, with later harvests for cold-region garlic [26,80]. Suitable drying weather matters because summer monsoon rain can increase decay [81]. Labor availability and market requirements also constrain horticultural harvest operations [82]. Here, the common 15 May date controlled the production calendar. It was not each genotype’s physiological maturity or a harvest recommendation for every region. Ranking changes persisted within a week of this date, but some trait and cultivar responses changed (Section 3.7). These tests assess sensitivity to the evaluation date without aligning developmental stages. Greater rank agreement toward July therefore does not establish comparable performance at maturity or suitability for later commercial harvest (Figure 8A).

The largest relative yield increases occurred for warm-region backgrounds in currently unfavorable cool environments (Figure 7). Northern sites already support cold-region garlic. Low current bulb mass and different progress by the common harvest amplify these ratios, suggesting improved suitability for the tested backgrounds rather than several-fold commercial gains [23]. Responses were smaller in established warm-region environments, particularly maritime Jeju. For context, CO_2_ enrichment increased mature onion yield by 29–51%, while warming reduced it [83]. Across C_3_ crops in field enrichment experiments, approximately 200 ppm enrichment increased yield by about 18% without resource stress [84]. Neither comparison validates the magnitude of the combined future response here.

Because the inputs represent average seasonal conditions rather than individual weather years, the results describe responses to representative climates, not to interannual variability or extreme weather. Rank persistence therefore means agreement across the climate states tested here. Future studies using weather data for individual years could examine how variability and extreme events affect these rankings [85].

### 4.6. Model Scope

Comparing two cultivar backgrounds tests retained parameters within one model, not structural uncertainty. Different models can project different climate responses and ideotypes [41,42,48]. The sufficient chilling assumption also leaves open whether future winters alone would support normal bulb development. Cultivar measurements of chilling responses, clove differentiation, and secondary growth would allow this assumption to be relaxed and bulb quality to be considered.

Resource feedbacks remain a priority for model development. Atmospheric dryness affects stomatal conductance and leaf energy balance, but soil water depletion does not feed back on stomatal closure. Garlic experiments have shown photosynthetic acclimation to elevated CO_2_, strong nitrogen effects, and no significant biomass response to enrichment [86]. These acclimation and nutrient feedbacks were not represented here. Canopy light interception and organ dry matter measurements can constrain carbon gain [87], while nitrogen experiments can test canopy photosynthesis and yield responses [88]. Integrating soil water and nitrogen would then enable rainfall, irrigation, and fertilization scenarios [32].

Parameter assumptions also affect cultivar comparisons. Leaf allometry remained fixed within each background. The virtual $V_{c,25}$ range centered on a published hardneck garlic estimate of 108.4 μmol m^−2^ s^−1^ [51], above the capacities of the two cultivar references and some other garlic measurements [86,89]. These higher simulated yields describe what the model predicts when greater photosynthetic capacity is combined with other traits. Whether such combinations can be developed through breeding and deliver similar gains over Namdo or Daeseo requires field testing.

## 5. Conclusions

Virtual genotype experiments showed that current performance alone is not a sufficient guide to future garlic improvement. Useful trait combinations varied among environments. Maritime comparisons suggested slower initiation and longer expansion as a hypothesis for testing. Long leaves with slower leaf tip appearance were favored in the southern mainland, while northern environments favored faster leaf initiation with slower appearance. The contrasting Namdo and Daeseo responses likewise support reassessing current cultivar choices against future regional conditions rather than assuming that present success ensures future suitability. Breeding should consider cultivar traits and planting time together for each target environment. Regional field phenotyping and model extensions for cultivar development and resource feedbacks can help translate these hypotheses into practical breeding targets.

## Figures and Tables

**Table 1:** Traits varied among the virtual garlic genotypes.

Trait	Symbol	Unit	Virtual Range
Initial leaf number	$N_{0}$	leaves	4, 5, 6, 7, 8
Maximum leaf initiation rate	$r_{i}$	d^−1^	0.07–0.14
Critical photoperiod for floral initiation	$P_{c}$	h	11.0–13.5
Developmental optimum	$T_{o}$	°C	20.0–24.5
Optimum to ceiling interval	$\Delta T_{c}$	K	10.0–14.0
Maximum leaf tip appearance rate	$r_{a}$	d^−1^	0.35–0.90
Potential longest leaf length	$L_{m}$	cm	55–115
Leaf expansion duration	$\tau_{e}$	d	7–21
Senescence threshold multiplier	$s_{g}$	–	1.0–4.0
Leaf angle factor	$f_{\theta }$	–	0.50–0.90
Carboxylation capacity	$V_{c,25}$	μmol m^−2^ s^−1^	90–125
Electron transport ratio	$\phi_{J}$	–	1.40–1.75
Stomatal slope	$g_{1}$	–	5.5–8.0

**Table 2:** Principal trait and process differences between the representative Namdo and Daeseo backgrounds.

Trait	Symbol	Unit	Namdo	Daeseo
Initial leaf number	$N_{0}$	leaves	8	8
Maximum leaf initiation rate	$r_{i}$	d^−1^	0.100	0.140
Maximum leaf tip appearance rate	$r_{a}$	d^−1^	0.679	0.800
Potential longest leaf length	$L_{m}$	cm	100.0	69.25
Maximum leaf elongation rate	$r_{e}$	cm d^−1^	6.12	6.50
Senescence threshold multiplier	$s_{g}$	–	1.29	4.00
Leaf area multipliers	$a_{1},a_{2}$	–	0.325, 2.0	0.600, 1.0
Peak leaf rank ratio	$q_{p}$	–	0.92	0.88
Maintenance respiration	$r_{m}$	g g^−1^ d^−1^	0.005	0.010
Synthesis efficiency	$y_{g}$	–	0.75	0.65
Carboxylation capacity	$V_{c,25}$	μmol m^−2^ s^−1^	50.23	49.99
Dark respiration at 25 °C	$R_{d,25}$	μmol m^−2^ s^−1^	1.67	2.26
Bulb allocation before/after scape appearance	$p_{b}$	fraction	0.45, 0.70	0.60, 0.80

**Table 3:** Study sites ordered from north to south with current crop season climate and production context. Coordinates use the World Geodetic System 1984. $\bar{T}$ is mean air temperature, $\bar{\mathrm{VPD}}$ is mean vapor pressure deficit, and $\sum R_{s}$ is cumulative solar radiation from 1 September to 15 May.

Site	Lat.	Lon.	$\bar{T}$ (°C)	$\bar{\mathrm{VPD}}$ (kPa)	$\sum R_{s}$ (MJ m^−2^)	Current Production Context
Chuncheon	37.903	127.736	7.0	0.42	2966	Cold inland reference
Danyang	36.986	128.367	7.3	0.41	2786	Cold-region ecotype
Seosan	36.777	126.494	7.9	0.40	2948	Cold-region ecotype
Uiseong	36.356	128.689	7.5	0.53	3040	Cold-region ecotype
Uiryeong	35.323	128.288	9.6	0.51	3323	Warm-region ecotype
Mokpo	34.817	126.382	10.5	0.35	2972	Warm-region ecotype
Namhae	34.817	127.926	10.9	0.42	3216	Warm-region ecotype
Goheung	34.618	127.276	10.0	0.47	3476	Warm-region ecotype
Haenam	34.554	126.569	9.6	0.47	3139	Warm-region ecotype
Jeju	33.294	126.166	12.9	0.29	2752	Maritime

**Table 4:** Trait values of three selected virtual genotypes. The leading profile ranked first in the southern mainland when sown on day 250 under both cultivar backgrounds in every climate state. The southern leader refers to current genotype rankings averaged across sowing dates and was the same in both backgrounds. The northern leader refers to the current leading profile in the Daeseo background at sowing day 250. Symbols and units follow Table 1.

Trait	Symbol	Leading Profile	Southern Leader	Northern Leader
Initial leaf number	$N_{0}$	6	5	4
Maximum leaf initiation rate	$r_{i}$	0.1211	0.0981	0.1146
Critical photoperiod	$P_{c}$	11.215	11.667	11.141
Developmental optimum	$T_{o}$	23.625	20.576	23.016
Optimum to ceiling interval	$\Delta T_{c}$	13.768	13.503	12.806
Maximum leaf tip appearance rate	$r_{a}$	0.6871	0.8394	0.8454
Potential longest leaf length	$L_{m}$	108.51	99.54	91.46
Leaf expansion duration	$\tau_{e}$	13.42	16.47	9.42
Senescence threshold multiplier	$s_{g}$	2.337	3.481	3.826
Leaf angle factor	$f_{\theta }$	0.8448	0.6901	0.7636
Carboxylation capacity	$V_{c,25}$	123.41	112.48	119.13
Electron transport ratio	$\phi_{J}$	1.662	1.410	1.554
Stomatal slope	$g_{1}$	6.441	6.489	6.622

## Data Availability

The model is available in the Garlic.jl GitHub repository at https://github.com/cropbox/Garlic.jl (accessed on 28 September 2026). Climate data may be requested from the provider under its access conditions.
